# Detrusor Underactivity as an HCN-Mediated Failure of Resilience in Aging

**DOI:** 10.1093/geroni/igaa057.431

**Published:** 2020-12-16

**Authors:** Dawn Rosenberg, Cara Hardy, Ramalakshmi Ramasamy, Phillip Smith

**Affiliations:** 1 UConn Health, Farmington, Connecticut, United States; 2 UConn Health, Farminton, Connecticut, United States

## Abstract

Sympathetic relaxation of the bladder wall permits low pressure urine storage and allows central regulation of afferent sensitivity to volume. Impaired regulation of volume sensitivity has been linked to symptoms of underactive bladder and cystometric detrusor underactivity. Hyperpolarization-activated cyclic nucleotide-gated channels (HCN channels) are mediators of sympathomimetic-induced detrusor relaxation in young mouse bladder tissue, however in bladder strips from old female mice, HCN blockade enhanced age-diminished isoproterenol-induced relaxation. We therefore hypothesized that loss of HCN would compromise cystometric function and enhance sympathomimetic responses in old mice. Male HCN1 KO mice (20-22 mo) and their WT littermates underwent pressure-flow cystometry under urethane anesthesia to assess urinary performance at the level of the autonomic nervous system in the absence of cortical control. Following cystometry, bladders were harvested and pharmacomyography was performed on bladder strips to determine tissue-level changes in the absence of CNS input. All mice responded to continuous-fill cystometry by establishing regular filling/voiding cycles. HCN KO mice function showed discrete changes in volume sensitivity vs. WT. Bladder strip studies showed minimal response to isoproterenol regardless of HCN status, and no significant differences in response to carbachol based on HCN status. We conclude that HCN status impacts the brainstem-bladder reflexic control over urine storage/voiding, but not by regulating bladder wall tensions during filling. The absence of HCN influence on the loss of end-organ responsiveness to sympathetic control in old mice points to an increasing dependency on central control mechanisms with aging.

